# Integrated surveillance in Kilifi reveals continued SARS-CoV-2 circulation and immune escape of emerging JN.1 sublineages post-pandemic

**DOI:** 10.3389/fcimb.2026.1910106

**Published:** 2026-09-03

**Authors:** Gloria Konyino, Bernadette Kutima, Sharon Owuor, Donwilliams Omuoyo, Gerald Waweru, Igor Andrade Santos, Arnold W. Lambisia, Esther Nyadzua Katama, Angela Maina, Joyce Uche Nyiro, Charles Agoti, Charles Sande, Philip Bejon, Dalan Bailey, George Mbugua Warimwe, L. Isabella Ochola-Oyier, Doreen Lugano, Steven Ger Nyanjom, James Nyagwange

**Affiliations:** 1 KEMRI-Wellcome Trust Research Programme, Center for Geographic Medicine Research, Coast, Kilifi, Kilifi County, Kenya; 2 Jomo Kenyatta University of Agriculture and Technology, Nairobi, Kenya; 3 Viral Glycoproteins Group, The Pirbright Institute, Pirbright, United Kingdom; 4 Nuffield Department of Medicine, Oxford University, Oxford, United Kingdom

**Keywords:** immune escape, JN.1 sub-lineages, low middle income countries, neutralizing antibodies, SARS-CoV-2, sub-Saharan Africa, viral evolution, whole genome sequencing

## Abstract

**Introduction:**

SARS-CoV-2 has been deprioritized post-pandemic, especially in Africa. Vaccination in Kenya was conducted using the Wuhan-based vaccines only, which showed reduced neutralization efficacy against Omicron and its sublineages.

**Methods:**

We performed SARS-CoV-2 immune surveillance in five health facilities within the Kilifi Health Demographic and Surveillance System from November 2024 to March 2026. Patients in the facilities presenting acute respiratory symptoms were recruited and nasopharyngeal/oropharyngeal swabs taken for SARS-CoV-2 RT-PCR. Convalescent plasma from 30 patients with confirmed SARS-CoV-2 RT-PCR positive tests and genome sequences were taken for sero-neutralization assays against the infecting variants LF.7, MV.1, and globally predominant strains, XEC.4, LP.8.1 and XFG.

**Results:**

In total 5115 patients were tested and 171 patients (3.4%) were positive for SARS-CoV-2. Cough (91.7%), nasal discharge (74.5%) and fever (55.4%) were the most common symptoms while wheezing (2%) and crackles (2.8%) were rarest. Sequencing revealed circulation of JN.1 omicron sub-lineages, LF.7, MV.1 and XEF. Sera from majority of individuals infected by the dominant variants, LF.7 and MV.1 were able to neutralize infecting variants but not the recently circulating XFG variant 23% (7/30) suggesting that infection with a JN.1 lineage virus alone is not sufficient to prevent re-infection with XFG. Overlapping titer distributions was observed between vaccinated and unvaccinated sera, with no statistically significant difference observed for any variant.

**Conclusion:**

Our study demonstrates that SARS-CoV-2 continues to circulate in coastal Kenya causing predominantly mild upper respiratory illness while evolution enabling immune escape. These findings emphasize the importance of integrated genomic and immunological surveillance to track emerging variants, evaluate population-level protection, and inform future vaccine update strategies and public health preparedness efforts.

## Introduction

SARS-CoV-2 continues to circulate globally in the post-pandemic period, yet surveillance has substantially declined across Sub-Saharan Africa (SSA) following the World Health Organization (WHO) declaration of the end of the COVID-19 pandemic in May 2023 (World Health Organization, 2023). There are critical gaps in understanding the ongoing burden of symptomatic disease in low- and middle-income settings. Ethiopia identified ongoing SARS-CoV-2 transmission among influenza-like illness and severe acute respiratory illness cases through 2023 and 2024, demonstrating the continued value of facility-based surveillance in characterizing respiratory virus epidemiology (Tayachew et al., 2026).

The Omicron variant, first detected in November 2021, has undergone continuous and rapid diversification under intense selective pressure from population immunity, generating successive sublineages that accumulate spike protein mutations conferring advantages in transmissibility and antibody evasion (Cerqueira-Silva et al., 2023; Ghazvini and Keikha, 2023; Scheaffer et al., 2023; Zhang et al., 2023; Machkovech et al., 2024). Between 2022 and 2023, SARS-CoV-2 underwent substantial antigenic evolution, marked by the successive emergence of Omicron sublineages (Dhawan et al., 2022; Liu et al., 2022; Ou et al., 2022; Wang et al., 2022). Early Omicron subvariants, including BA.1, BA.2, and their descendants, displaced Delta (B.1.617.2) globally within weeks of emergence, largely due to their substantially increased resistance to neutralization by vaccine-elicited and natural-infection-induced antibodies (Dhawan et al., 2022; Shah and Woo, 2022; Tang et al., 2023). In early 2023, the XBB sublineage gained global dominance owing to its enhanced capacity to evade neutralizing antibodies, prompting a worldwide transition in vaccine antigen composition to monovalent XBB.1.5 formulations (Wang et al., 2023; Zhang et al., 2023; Planas et al., 2024). Shortly thereafter, the BA.2.86 sublineage emerged, bearing more than 30 spike mutations compared with its predecessor and demonstrating significant immune evasion (Sheward et al., 2023; Planas et al., 2024). By the end of 2023, BA.2.86 acquired the spike mutation L455S, giving rise to the dominant lineage JN.1. Subsequent JN.1 sublineages, including LP.8.1.1 and XFG, accumulated additional mutations such as F456L and R346T that further enhanced viral transmission and immune evasion (Kaku et al., 2024b; Li et al., 2024b; Wang et al., 2024). JN.1 and its descendants, LP.8.1.1 and XFG, remain prevalent and confer mutations associated with substantial immune escape and have been shown to reduce neutralization by several monoclonal antibodies (Klinakis et al., 2021; Li et al., 2024b; Wang et al., 2024). Consequently, in 2025, the WHO, FDA, and EMA issued directives for changes in booster vaccines to monovalent JN.1 and its derivatives KP.2, and LP.8.1 and recently, in May 2026, updated the vaccine regimen to include XFG antigen, which has been shown to induce high-neutralizing antibody titers against other JN.1 descendants variants (U.S. Food and Drug Administration, 2025; European Medicines Agency, 2026; European Medicines Agency, 2026; World Health Organization, 2026).

In SSA, COVID-19 vaccine coverage has remained critically low. As of December 2023, only 32% of the WHO African Region population had completed a primary vaccine series, and just 21% had received any booster dose, whose formulations were based on ancestral Wuhan-1 spike protein, with no JN.1 updated vaccine having been deployed across the region (Doshi et al., 2021). Due to the rapid evolution of SARS-CoV-2, particularly within the Omicron lineage, vaccines originally developed based on the ancestral Wuhan-Hu-1 strain in 2021 exhibited reduced neutralization against later sublineages, including JN.1 and its derivatives (Catanzaro et al., 2020; Ema, 2026). While asymptomatic infection predominates in coastal Kenya (Nyagwange et al., 2022; Bejon et al., 2025), this study demonstrates that symptomatic SARS-CoV-2 infections continue to occur, underscoring a quiet and underrecognized burden of disease in the post-pandemic period as priorities have shifted away from COVID-19 surveillance. We have previously shown seasonal annual patterns of SARS-CoV-2 in coastal Kenya and a decline in both natural and vaccine-induced antibody protection with the emergence of new Omicron variants in Kenya, including JN.1, and demonstrated the need for continued genomic and immune surveillance to protect public health and improve vaccine and therapeutic strategies in the country and globally (Lugano et al., 2024; Lambisia et al., 2026). Here we incorporate immune surveillance, revealing not only the continued circulation of SARS-CoV-2 but also the immune escape of emerging JN.1 lineages, particularly XFG, from preexisting immunity.

## Methods

### Study design and participants

This study was approved by the Scientific and Ethical Review Unit (SERU) at the Kenya Medical Research Institute (KEMRI/SERU/CGMR-C/4807 and 3103). This was a prospective-health-facility-based observational study with a single follow-up conducted to patients with SARS-CoV-2 variants of interest in Kilifi. Patients presenting with acute respiratory symptoms at five local health facilities, Kilifi County Hospital (KCH), Matsangoni Health Centre, Mavueni Health Centre, Mtondia Health Centre, and Pingilikani Health Centre, within the KHDSS were enrolled in the study between November 2024 and March 2026 and screened for SARS-CoV-2 by RT-PCR. Demographic information such as age, sex, clinical symptoms, vaccination history, and site of recruitment was recorded at enrollment. Convalescent plasma from 30 patients with confirmed SARS-CoV-2 RT-PCR positive tests and genome sequences were taken for sero-neutralization. The participants were approached sequentially as they presented for follow-up, and only those who were willing and able to provide a serum sample under informed consent were included. No further selection criteria (e.g., age, sex, severity) were applied. No random sampling was performed.

### Nasopharyngeal sample collection and SARS-CoV-2 screening

Nasopharyngeal and oropharyngeal swabs were collected from eligible individuals in viral transport media. The collected specimens were triple-packed and transported in cold chain (4°C) to the KEMRI-Wellcome Trust Research Programme (KWTRP). Total nucleic acids were extracted and screened for SARS-CoV-2 using real-time RT-PCR as described before (Lambisia et al., 2025a; Lambisia et al., 2026). Briefly, primers/probes targeting the envelope (E) gene (forward: 5′-ACA GGT ACG TTA ATA GTT AAT AGC GT-3′, reverse: 5′-ATA TTG CAG TAC GCA CAC A-3′ and probe 5′-ACA CTA GCC ATC CTT ACT GCG CTT CG-3′) were used in RT-PCR. For the multiplex PCR, we used QIAGEN Multiplex RT-PCR + R Kit (Qiagen, UK). We included both negative and positive controls from the start of RNA extraction to RT-PCR steps in all reactions. SARS-CoV-2 samples within a cycle threshold (Ct) value <35.0 were selected for sequencing.

### Whole-genome sequencing and phylogenetic analysis

Total RNA was re-extracted from 140 µL samples that tested positive using the QIAamp Viral RNA Mini Kit (Qiagen, Manchester, UK) following the manufacturer’s protocol as mentioned before (Lambisia et al., 2025a; Lambisia et al., 2026). Briefly, 8 µL of RNA was reverse-transcribed using 2 mL of LunaScript RT Mix (NEB, E3010, MA, USA) by incubating at 25°C for 2 min, 55°C for 10 min, and 95°C for 10 min. The cDNA was then amplified by multiplex PCR using ARTIC primers V5.3.2 in 2 reactions. Then, 1.3 µL of cDNA, 2 μL of primer pools, 6.3 μL of Q5 Hot Start High-Fidelity 2X Master Mix (NEB M0494, MA, USA), and 1.9 μL of nuclease-free water were combined in the reaction under the following thermocycling conditions: 1 cycle of 98°C for 30 s, followed by 25 cycles of 98°C for 30 s and 65°C for 5 min, 15 cycles of 62.5°C for 5 min and 98°C for 15 s and 1 cycle of 62.5°C for 5 min. The two reactions were then pooled and cleaned using 1X AMPure XP beads (Beckman Coulter, A63881, Indianapolis, IN, USA) (Quick, 2024). The resulting amplicons were end-repaired and ligated to Oxford Nanopore barcoded adapters using the SQK-LSK114 kit, followed by a final cleanup and library quantification (qubit concentrations of 15 ng/µL) prior to sequencing on a GridION device using R9.4.1 flow cells (Lugano et al., 2024). Data assembly was done using the ARTIC Network v4 amplicon protocol. Raw reads were base-called and demultiplexed with artic guppylex module. Viral reads were quality-filtered (Phred ≥20) and trimmed to remove adapters and then aligned to the Wuhan-Hu-1 reference (MN908947.3) using minimap2 v2.17. The alignments were sorted and indexed with SAMtools v1.10; unmapped reads were removed. Primer sequences were hard-clipped using iVar v1.4.2 (ivar trim -e) guided by the ARTIC nCoV-2019 v5 primer scheme (GitHub: primer_schemes/nCoV-2019/V5.3.2). Variants relative to the reference were called using SAMtools mpileup and BCFtools v1.10 with a minimum depth of 10× and minimum base quality of 20. Additional quality metrics were computed with SAMtools coverage, iVar, and QUAST v5.2. Lineage assignment was performed using Pangolin (v4.4). Sequencing success rates were calculated as the number of samples yielding genomes meeting our quality thresholds (≥90% completeness and ≥10× mean coverage) divided by the total number of samples sequenced in each Ct category and then multiplied by 100. For the phylogenetic analysis, we selected 317 publicly available SARS-CoV-2 genomes from the GISAID platform that were sampled between 2023 and 2025 from 28 countries. Only genomes with ≥90% completeness, <5% Ns, and ≥10× mean coverage were retained. A total of 30 additional genomes generated in this study were yielding a final data set of 347 sequences. The genomes were aligned using MAFFT v7.526 (Galaxy version 7.526+galaxy2) with the --globalpair --maxiterate 1000 options, reference-guided to the Wuhan-Hu-1 sequence (MN908947.3). The tree was inferred with 1,000 ultrafast bootstrap replicates with bias correction for SNP data. The resulting tree was visualized and annotated in iTOL (Interactive Tree Of Life), with nodes colored by lineage and sampling country (Minh et al., 2020; Kalyaanamoorthy et al., 2017).

### Pseudovirus neutralization assay

Pseudovirus neutralization assays were performed against a panel of SARS-CoV-2 lineages selected to reflect both local and global epidemiological relevance. Locally circulating lineages LF.7 and MV.1 were included to evaluate neutralizing antibody responses against variants currently predominant in our region. In addition, we selected globally prevalent lineages XEC.4, LP.8.1, and XFG, as these had been reported as the most abundant circulating variants worldwide during the study period. Neutralizing antibody titers against the selected variants, JN.1 (comparator), LF.7, MV.1, XEC, LP.8.1, and XFG, were measured using pseudovirus neutralization assays. The pseudovirus production and assay validation have been described before (Nyagwange et al., 2023) Briefly, a lentiviral expression system was used to produce pseudoviruses for Omicron sublineages JN.1, LF.7, MV.1, XEC, LP.8.1, and XFG. Three plasmids, coding for the MLV-gag/pol backbone, luciferase, and full-length spike protein of the different variants, were co-transfected into HEK293T cells using PEI (cat. no. 24765, Polysciences) to produce a single round of infection-competent pseudoviruses. The media were changed 24 h post-transfection, and the pseudovirus was harvested 72 h post-transfection. The pseudoviruses were aliquoted and frozen for long-term storage at -80°C. Relative light units (RLU) were measured to quantify luminescence intensity as the readout of lentiviral pseudovirus neutralization. Virus infectivity of the variants was determined by titration on HEK293T (hACE2-hTMPRSS2)-stable cells, and pseudovirus dilutions giving >20,000 RLU were selected for the assay. To test for neutralization, 50 μL of virus was immediately mixed with 50 μL of serially diluted (2×) serum and incubated for 1 h at 37°C. Following the incubation, 10,000 HEK293T (hACE2-hTMPRSS2) cells/well (in 100 μL of media) were directly added to the antibody–virus mixture. The plates were then incubated at 37°C for 72 h. To check for neutralization, HEK293T (hACE2-hTMPRSS2) cells were lysed using a lysis buffer (cat. no. E2661, Promega). Luciferase intensity was then read on a luminometer with luciferase substrate according to the manufacturer’s instructions (cat. no. E2650, Promega). The percentage of neutralization was calculated using the following equation: 100 × (1 − (RLU of sample − average RLU of background)/average RLU of virus only − average RLU of background)), where the background was cell-only control. To determine the ID_50_ value, a dose–response curve model was fitted using the sample dilution and the corresponding neuralization percentage.

### Statistical analysis

Sero-neutralization rates were calculated as the proportion of patients with ID_50_ >50, a threshold previously associated with 50% neutralization against SARS-CoV-2 infection (Khoury et al., 2021). The calculation of ID_50_ has been described previously (Nyagwange et al., 2023). Wilson exact 95% confidence intervals were used (Wilson, 1927). Geometric mean titer (GMT) fold reductions relative to JN.1 reference were calculated as GMT(JN.1)/GMT (variant) for each of the five variants. Geometric mean titers (GMT) were computed from log-transformed ID_50_ values including only samples above the lower limit of detection (Reverberi, 2008). GMT fold reductions vs. JN.1 was calculated from 2,000 bootstrap resamples. Group comparisons used paired Wilcoxon signed-rank tests with Benjamini–Hochberg correction for multiple testing across six simultaneous variant comparisons (Benjaminit and Hochberg, 1995). A twofold GMT reduction from JN.1 was used as the threshold for meaningful immune escape, consistent with the influenza antigenic studies (Wilks et al., 2023). Paired Wilcoxon signed-rank tests were used to compare log10-transformed ID_50_ values against each variant relative to the JN.1 reference. All five comparisons were conducted simultaneously, and raw *p*-values were adjusted for multiple testing using the Benjamini–Hochberg false discovery rate (FDR) procedure. We also aimed to ascertain whether prior COVID-19 vaccination with ancestral-based Wuhan vaccines influenced the magnitude of convalescent neutralizing antibody responses against Omicron lineages following natural infection and hence stratified the patients by COVID-19 vaccination status, which resulted in two groups: vaccinated 33% (10/30) and unvaccinated 60% (18/30). Differences in neutralization titers between vaccinated and unvaccinated patients were assessed for each variant separately using the Wilcoxon rank sum test with statistical significance set at *p* < 0.05.

Data analysis was run on R v4.5.3 and RStudio 2025.05.0 + 496.

## Results

### Health facility surveillance

**Figure 1:**
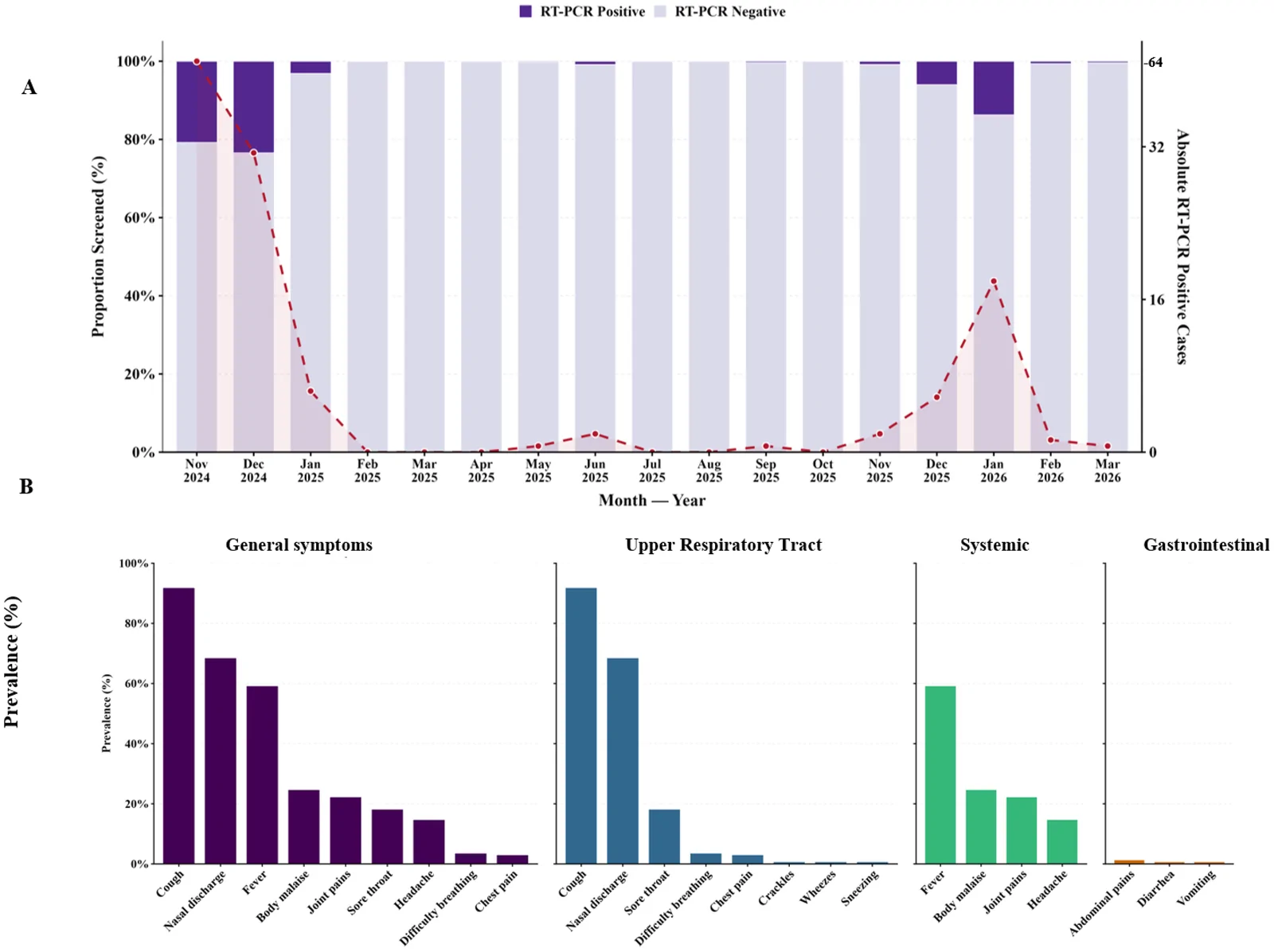
Monthly SARS-CoV-2 screening and symptom prevalence across five health facilities within the KHDSS. **(A)** Light purple bars represent the overall proportion of patients who were tested, and dark purple bars represent the proportion who tested RT-PCR positive for SARS-CoV-2. The red dashed line with points represents the absolute number of RT-PCR positive cases per month, plotted on a linear axis (right Y-axis). The secondary Y-axis (right) displays the absolute positive case counts (0–64 cases) aligned to the red line. **(B)** Symptoms are presented across four panels—general symptoms, upper respiratory tract, systemic, and gastrointestinal—with each panel displaying the percentage of SARS-CoV-2-positive patients reporting each symptom.

Out of the 5,115 patients screened, 3.4% (171/5115) tested SARS-CoV-2 positive by RT-PCR (**Table 1**). The highest SARS-CoV-2 prevalence occurred in November 2024 at 22.7% (64/308) and December 2024 at 23.5% (49/209). Case detection declined sharply, thereafter peaking again in December 2025 at 6% (9/150) and January 2026 at 13.7% (28/204) (**Figure 1A**). Among the 171 SARS-CoV-2-positive participants, upper respiratory tract symptoms dominated the clinical presentation (**Figure 1B**). Cough was the most prevalent symptom reported at 91.8% (157/171), followed by nasal discharge at 68.4% (117/171) and fever at 59.1% (101/171). Symptoms associated with severe SARS-CoV-2, such as difficulty in breathing 3.5% (6/171) and wheezing 0.6% (1/171), were also reported among the cohort.

**Table 1:** Baseline characteristics of patients presenting with acute respiratory symptoms across five health facilities within the Kilifi Health and Demographic Surveillance System (KHDSS).

Characteristic	Overall patients tested (*n* = 5,115)	SARS-CoV-2 positive (*n* = 171)	SARS-CoV-2 negative (*n* = 4,944)
Age, median (IQR), years	18.3 (5.9 to 32.9)	24.9 (14 to 46.6)	18.1 (5.7 to 32.6)
Age group, *n* (%)			
<18	2,450 (47.9%)	55 (32.2%)	2,395 (48.4%)
18–40 years	1,592 (31.1%)	62 (36.3%)	1,530 (30.9%)
41–60 years	520 (10.2%)	29 (17%)	491 (9.9%)
>60	453 (8.9%)	25 (14.6%)	428 (8.7%)
Sex, *n* (%)			
Female	3,133 (61.3%)	115 (67.3%)	3,018 (61%)
Male	1,883 (36.8%)	56 (32.7%)	1,827 (37%)
Health facility, *n* (%)			
Kilifi County Hospital	1,142 (22.3%)	31 (18.1%)	1,111 (22.5%)
Matsangoni	987 (19.3%)	32 (18.7%)	955 (19.3%)
Mavueni	1,035 (20.2%)	42 (24.6%)	993 (20.1%)
Mtondia	980 (19.2%)	39 (22.8%)	941 (19%)
Pingilikani	971 (19%)	27 (15.8%)	944 (19.1%)

### Characteristics of the convalescent cohort

Most participants were female (73%, 22/30), and the median age was 35.4 years (IQR, 25.7–46.1) (**Table 2**). Most patients were unvaccinated (60%, 18/30). The most prevalent symptom was cough (90.0%, 27/30), followed by nasal discharge (63.3%, 19/30).

**Table 2:** Baseline characteristics of the convalescent cohort.

	Health facility					
Characteristic	KCH (*n* = 6)	Matsangoni (*n* = 8)	Mavueni (*n* = 7)	Mtondia (*n* = 5)	Pingilikani (*n* = 4)	Total (*n* = 30)
Sex						
Male	4 (67%)	0 (0%)	1 (14%)	1 (20%)	2 (50%)	8 (27%)
Female	2 (33%)	8 (100%)	6 (86%)	4 (80%)	2 (50%)	22 (73%)
Age group						
<18	0 (0%)	0 (0%)	0 (0%)	2 (40%)	0 (0%)	2 (7%)
18–60 years	5 (83%)	6 (75%)	6 (86%)	3 (60%)	4 (100%)	24 (80%)
>60	1 (17%)	2 (25%)	1 (14%)	0 (0%)	0 (0%)	4 (13%)
COVID-19 vaccination						
Yes	5 (83%)	3 (38%)	2 (29%)	0 (0%)	0 (0%)	10 (33%)
No	1 (17%)	3 (38%)	5 (71%)	5 (100%)	4 (100%)	18 (60%)
Unknown	0 (0%)	2 (25%)	0 (0%)	0 (0%)	0 (0%)	2 (7%)
Infecting variant (WGS)						
LF.7	4 (67%)	7 (88%)	5 (71%)	5 (100%)	4 (100%)	25 (83%)
MV.1	2 (33%)	1 (13%)	1 (14%)	0 (0%)	0 (0%)	4 (13%)
XEF	0 (0%)	0 (0%)	1 (14%)	0 (0%)	0 (0%)	1 (3%)

### Genomics and phylogeny of the viruses in convalescent patients

**Figure 2:**
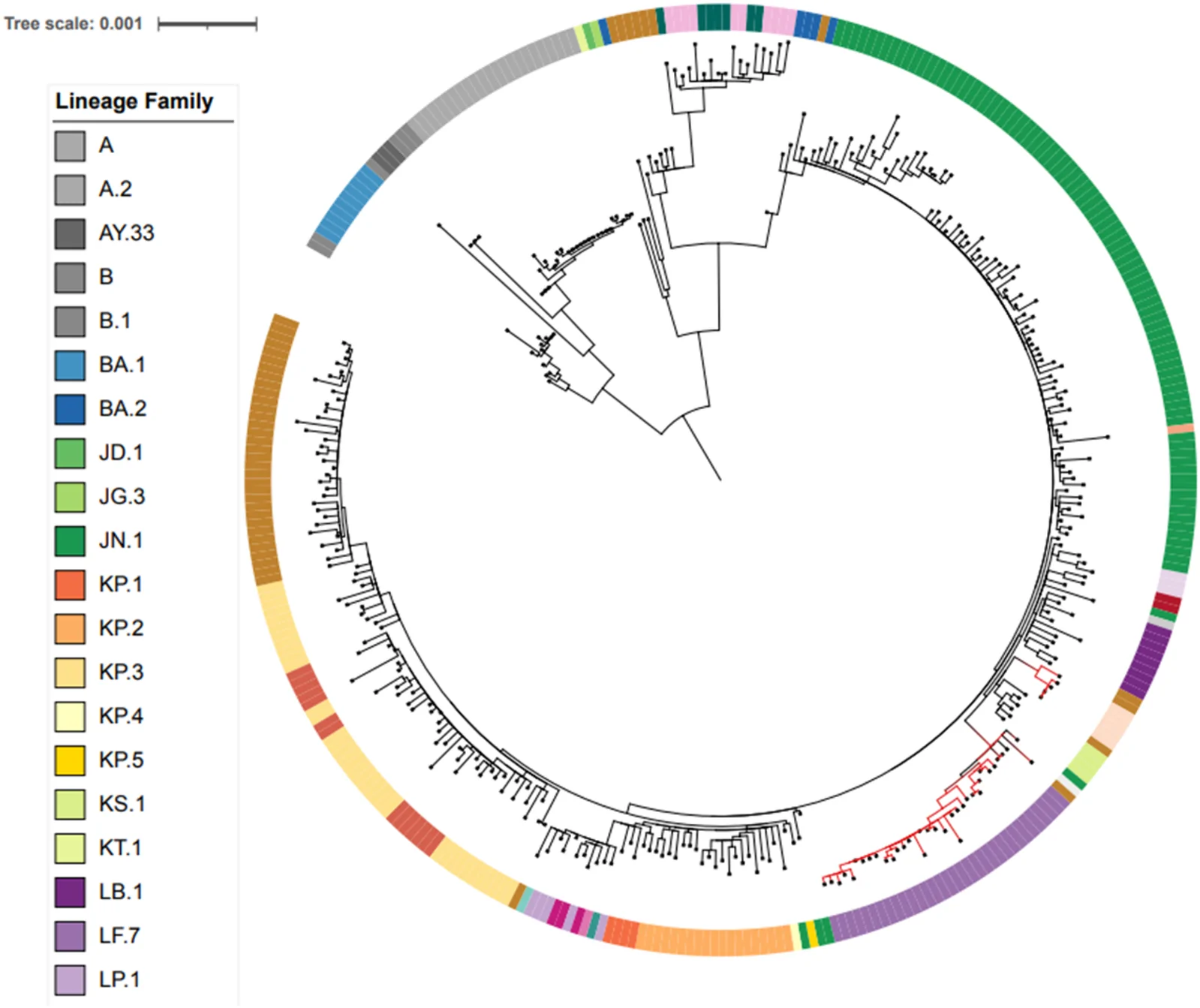
Maximum likelihood phylogenetic tree of 347 SARS-CoV-2 sequences comprising 37 Kenyan sequences and 310 global background sequences sampled across 28 countries (2023–2025). Branch lengths represent nucleotide substitutions per site (scale bar = 0.001). The color ring denotes PANGO lineage family. The red-colored branches denote Kenyan sequences. Tree visualization was performed using the Interactive Tree of Life (iTOL; itol.embl.de).

Our sequencing success rate was 96% (29/30), with all genomes yielding genome completeness of more than 92%. Whole-genome sequencing showed that LF.7 was the dominant infecting lineage (83.3%, 25/30), followed by MV.1 variant (13.3%, 4/30) and XEF (3.3%, 1/30), all classified within the JN.1 sublineage clade per PANGO nomenclature. (**Table 2**). Additionally, the XEF sequence identified from a sample with a Ct value of 33 showed 95.2% genome completeness (Nextclade) and sufficient coverage to support robust lineage assignment. This sequence was inferred to be a recombinant of LB.1.4 and KP.3 carrying the S: V1104L mutation, and detailed characterization is still emerging. We mapped these samples with globally available sequences on the Galaxy platform and demonstrated that indeed they are derivatives of JN.1, stemming away from other prominent sublineages such as KP.3, KP.3.1.1, and XEC (**Figure 2**).

### Sero-neutralization of JN.1 derivatives LF.7, MV.1, XEC.4, LP.8.1., and XFG

**Figure 3:**
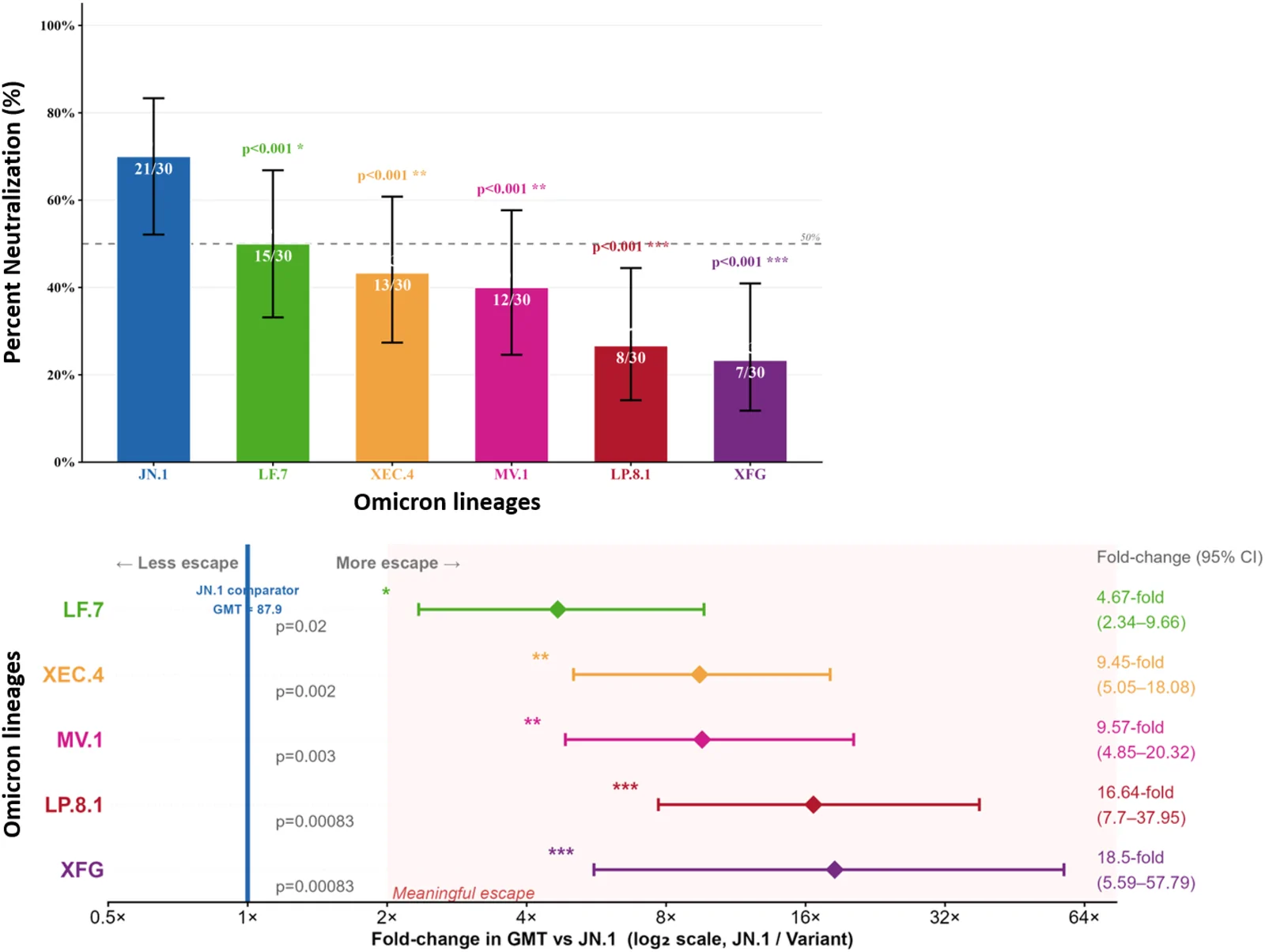
Neutralizing antibody responses and fold change of JN.1 and its sublineages. **(A)** Proportion of patients achieving sero-neutralization (ID_50_) for each variant. Error bars represent exact Wilson 95% confidence intervals. The dashed reference line at 50% denotes the cutoff neutralization ID_50_. **(B)** Forest plot showing the fold change in GMT (geometric mean titer) relative to JN.1. Diamond symbols indicate point estimates of GMT ratio; horizontal whiskers indicate 95% confidence intervals derived from 2,000 bootstrap resamples. The pink-shaded region (>2×) denotes the threshold for meaningful immune escape; p-values are annotated for each comparison (*p < 0.05, **p < 0.01, ***p < 0.001).

JN.1 sero-neutralization was 70.0% (21/30), reflecting cross-neutralizing responses from the predominantly LF.7-infected cohort against the parental lineage. Sero-neutralization rates against the remaining variants were LF.7 50.0% (15/30), XEC.4 43.3% (13/30), MV.1 40.0% (12/30), LP.8.1 26.7% (8/30), and XFG 23.3% (7/30) (**Figure 3A**). A fold reduction greater than 2 was used as the threshold for meaningful immune escape (**Figure 3B**) (Wilks et al., 2023). The geometric mean titer of JN.1 was 87. GMT fold reductions relative to JN.1 were statistically significant for all five variants after Benjamini–Hochberg correction (**Figure 3B**): LF.7, 4.67-fold (95% bootstrap CI 2.34–9.66, *p* = 0.02); XEC.4, 9.45-fold (5.05–18.08, *p* = 0.002); MV.1, 9.57-fold (4.85–20.32, *p* = 0.003); LP.8.1, 16.64-fold (7.70–37.95, *p* = 8.3 × 10^−4^); and XFG, 18.5-fold (5.59–57.79, *p* = 8.3 × 10^−4^).

**Figure 4:**
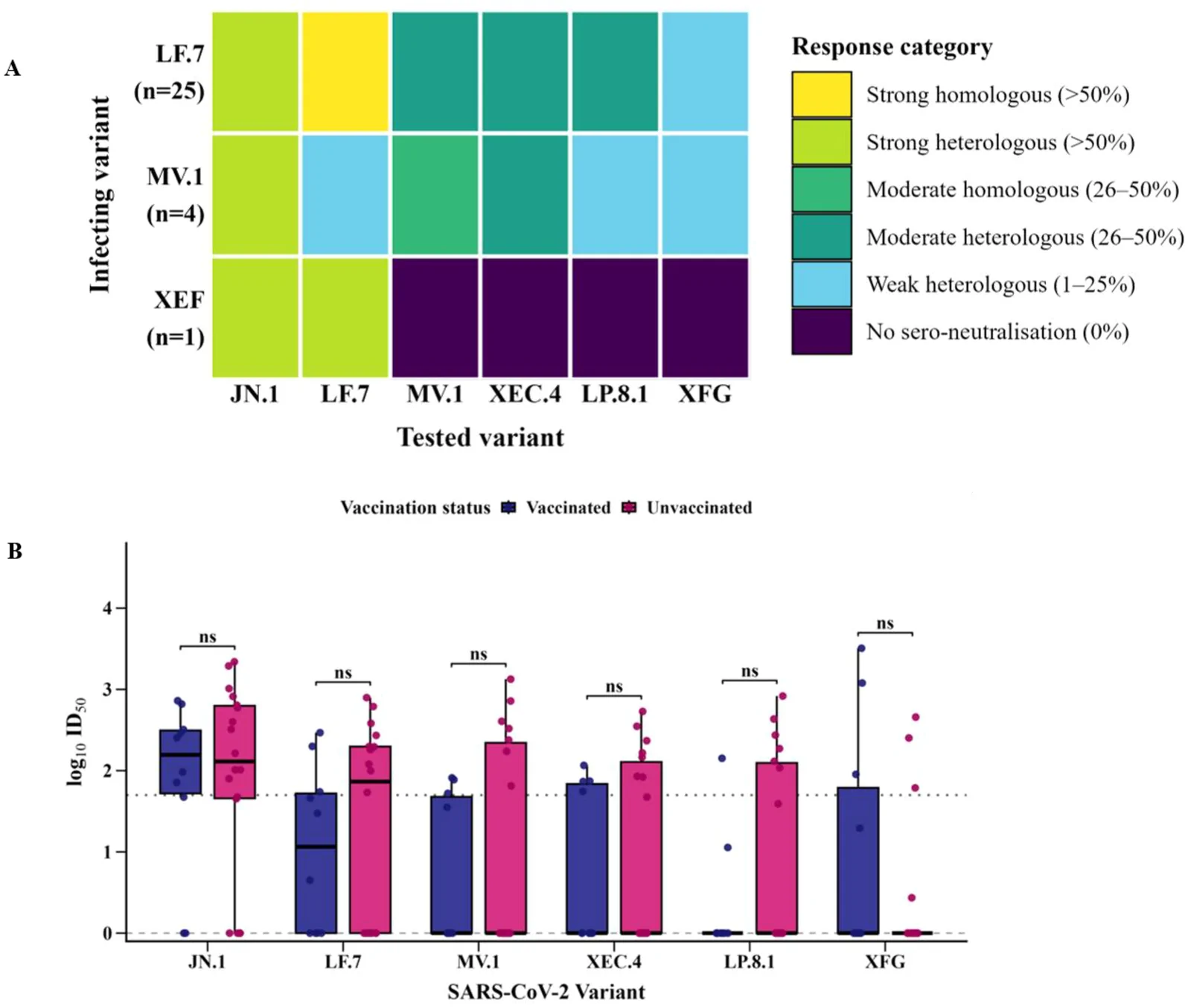
Cross-reactive neutralization of JN.1 variants stratified by infecting lineage and vaccination status. **(A)** Percentage of participants in each WGS-confirmed infecting variant group achieving sero-neutralization (ID_50_) against six JN.1-lineage. The cell color indicates the response category as homologous or heterologous. **(B)** Displays log_10_ ID_50_ neutralization titers stratified by vaccination status across all six variants, with boxplots showing median and interquartile range, individual data points, a seroprotection threshold (dotted line, ID_50_), and Wilcoxon rank-sum Benjamini–Hochberg-adjusted p-values annotated above each variant pair (ns = not significant; all p > 0.05).

Stratification by WGS-confirmed infecting variants revealed reduced cross-reactivity neutralizations to non-infecting variants (**Figure 4A**). The LF.7-infected participants (*n* = 25) showed a higher homologous LF.7 neutralization rate (52%, GMT = 142) than the MV.1-infected participants tested against LF.7 (25%, GMT = 96). The LF.7-infected participants also demonstrated broad cross-reactive neutralization against JN.1 (64%) and moderate responses against MV.1, XEC.4, and LP.8.1 (28%–44%), but low neutralization against XFG (24%). The single XEF-infected participant showed no detectable neutralization against XFG while achieving strong neutralizing responses against JN.1 and LF.7. Neutralization profiles across the five Omicron variants revealed overlapping titer distributions between vaccinated and unvaccinated groups with no statistically significant difference observed by any variant (**Figure 4B**). Both groups demonstrated high neutralizing titers against JN.1 and LF.7 but moderate and low titers against the remaining variants (MV.1, XEC.4, and LP.8.1.1).

## Discussion

Omicron JN.1 and its sublineages have been predominant since late 2023, necessitating continued genomic and immune surveillance to assess population immunity against SARS-CoV-2 infection and, in particular, the capacity of circulating antibodies to neutralize JN.1 sublineages and related Omicron variants (Li et al., 2024b; Lugano et al., 2024; Wang et al., 2024). In December 2025, the World Health Organization Technical Advisory Group on COVID-19 Vaccine Composition (TAG-CO-VAC) recommended that countries procure and deploy monovalent JN.1 lineage-adapted vaccine formulations (LP.8.1 composition); however, many countries in SSA have not updated the vaccine schedule accordingly (WHO, 2025). The first-generation COVID-19 vaccines deployed in Kenya in 2021 were based on the Wuhan-Hu-1 SARS-CoV-2 spike, with vaccine coverage as of December 2023 reaching only 32% (Lawal et al., 2022). This implies that the population immunity in SSA is dependent on naturally induced rather than vaccine-induced immunity (Lugano et al., 2024; Lambisia et al., 2025a; Lambisia et al., 2025b; Musundi et al., 2025).

Our findings demonstrate the continued circulation and evolution of SARS-CoV-2 within Kilifi during the 2024–2026 surveillance period, with 3.4% of patients presenting with acute respiratory symptoms testing positive for SARS-CoV-2. Compared to 2020–2021 when 7.6% symptomatic patients tested positive in the coastal region, 3.4% presents a relatively low prevalence, suggesting lower transmission intensity compared with earlier pandemic waves, which is likely due to increasing population immunity from prior infection and vaccination (Nyagwange et al., 2022; Musundi et al., 2025). The clustering of most cases in November–December 2024 indicates episodic transmission dynamics, consistent with the post-pandemic pattern of SARS-CoV-2 circulation driven by waning immunity and emergence of immune-evading variants (Lugano et al., 2024; Musundi et al., 2025). Clinical presentation was dominated by upper respiratory symptoms, particularly cough, nasal discharge, and fever, while severe symptoms such as difficulty in breathing and wheezing were uncommon, aligning with global observations that Omicron-descendent variants, including JN.1 lineages, are associated with milder upper respiratory disease and reduced pathogenicity compared with earlier variants (World Health Organization (WHO), 2024).

Genomic analysis showed an overwhelming predominance of LF.7, with smaller contributions from MV.1 and the recombinant XEF lineage, all within the JN.1 clade, with phylogenetic analysis confirming that Kenyan viruses clustered within the broader JN.1 evolutionary landscape but remained distinct from globally dominant sublineages such as KP.3 and XEC, emphasizing the importance of regional genomic surveillance (Kaku et al., 2024a; Li et al., 2024a; Lugano et al., 2024).

Neutralization assays demonstrated substantial antigenic drift with markedly reduced neutralization observed against LF.7, MV.1, XEC.4, LP.8.1, and especially XFG similar to other studies done on antigenic cartography (Abbad et al., 2026). All variants showed greater than twofold reductions in GMT relative to JN.1, indicating significant immune escape. The LF.7-infected patients demonstrated broad but incomplete neutralization with poor activity against XFG, indicating that newer variants may increasingly evade infection-induced immunity (Li et al., 2024b; Liu et al., 2025). The absence of a statistically significant difference in neutralizing antibody titers between vaccinated and unvaccinated patients can be attributed to the combined effects of waning of vaccine-induced immunity and limited cross-neutralization of variants that have accumulated extensive mutations in the spike protein relative to the ancestral Wuhan-Hu-1 strain (Reynolds et al., 2022; Torresi and Edeling, 2024). The tested lineages (LF.7, MV.1, XEC.4, LP.8.1, and XFG) carry numerous spike mutations in the receptor-binding domain and N-terminal domain that confer altered antigenicity and enhanced escape from antibodies elicited by ancestral-strain vaccines (Guo et al., 2025; Mellis et al., 2025).

A key limitation of this study is the restricted geographic scope which is limited to samples from hospitals in the KHDSS, reducing our findings to only one county compared to a nationwide assessment of population immunity against emerging SARS-CoV-2 JN.1 strains. Nevertheless, the study demonstrates the value of integrating clinical, genomic, and immune surveillance into routine public health systems and can serve as a national model to strengthen epidemic preparedness, monitor emerging variants, inform vaccine strategies, and build resilient surveillance systems.

In conclusion, this study reveals the ongoing but under-recognized symptomatic wave of SARS-CoV-2 transmission in coastal Kenya post-pandemic, underscoring the need for integrated surveillance and updated vaccine regimens for vulnerable populations.

## Data Availability

The datasets generated and analysed during the current study are available in the Havard Dataverse repository, https://doi.org/10.7910/DVN/Y0JX5E.
